# The Evolution of Life Modes in Stictidaceae, with Three Novel Taxa

**DOI:** 10.3390/jof7020105

**Published:** 2021-02-02

**Authors:** Vinodhini Thiyagaraja, Robert Lücking, Damien Ertz, Samantha C. Karunarathna, Dhanushka N. Wanasinghe, Saisamorn Lumyong, Kevin D. Hyde

**Affiliations:** 1 Department of Entomology and Plant Pathology, Faculty of Agriculture, Chiang Mai University, Chiang Mai 50200, Thailand; vino.thiyagaraja@gmail.com; 2 Centre of Excellence in Fungal Research, Mae Fah Luang University, Chiang Rai 57100, Thailand; 3 CAS Key Laboratory for Plant Biodiversity and Biogeography of East Asia (KLPB), Kunming Institute of Botany, Chinese Academy of Science, Kunming 650201, China; samanthakarunarathna@gmail.com (S.C.K.); dnadeeshan@gmail.com (D.N.W.); 4 Botanischer Garten und Botanisches Museum, Freie Universität Berlin, Königin-Luise-Str. 6-8, 14195 Berlin, Germany; R.Luecking@bgbm.org; 5 Research Department, Meise Botanic Garden, Nieuwelaan 38, BE-1860 Meise, Belgium; damien.ertz@jardinbotaniquemeise.be; 6 Fédération Wallonie-Bruxelles, Service Général de l’Enseignement Supérieur et de la Recherche Scientifique, Rue A. Lavallée 1, BE-1080 Bruxelles, Belgium; 7 World Agro forestry Centre East and Central Asia, Kunming 650201, China; 8 Department of Biology, Faculty of Science, Chiang Mai University, Chiang Mai 50200, Thailand; scboi009@gmail.com; 9 Center of Excellence in Bioresources for Agriculture, Industry and Medicine, Faculty of Science, Chiang Mai University, Chiang Mai 50200, Thailand; 10 Innovative Institute of Plant Health, Zhongkai University of Agriculture and Engineering, Haizhu District, Guangzhou 510225, China

**Keywords:** 3 new taxa, ancestral character state analysis, asexual morph, Lecanoromycetes, *Ostropomyces*, sexual morph, *Sphaeropezia*

## Abstract

Ostropales *sensu lato* is a large group comprising both lichenized and non-lichenized fungi, with several lineages expressing optional lichenization where individuals of the same fungal species exhibit either saprotrophic or lichenized lifestyles depending on the substrate (bark or wood). Greatly variable phenotypic characteristics and large-scale phylogenies have led to frequent changes in the taxonomic circumscription of this order. Ostropales *sensu lato* is currently split into Graphidales, Gyalectales, Odontotrematales, Ostropales *sensu stricto*, and Thelenellales. Ostropales *sensu stricto* is now confined to the family Stictidaceae, which includes a large number of species that are poorly known, since they usually have small fruiting bodies that are rarely collected, and thus, their taxonomy remains partly unresolved. Here, we introduce a new genus *Ostropomyces* to accommodate a novel lineage related to *Ostropa*, which is composed of two new species, as well as a new species of *Sphaeropezia*, *S. shangrilaensis*. Maximum likelihood and Bayesian inference analyses of mitochondrial small subunit spacers (mtSSU), large subunit nuclear rDNA (LSU), and internal transcribed spacers (ITS) sequence data, together with phenotypic data documented by detailed morphological and anatomical analyses, support the taxonomic affinity of the new taxa in Stictidaceae. Ancestral character state analysis did not resolve the ancestral nutritional status of Stictidaceae with confidence using Bayes traits, but a saprotrophic ancestor was indicated as most likely in a Bayesian binary Markov Chain Monte Carlo sampling (MCMC) approach. Frequent switching in nutritional modes between lineages suggests that lifestyle transition played an important role in the evolution of this family.

## 1. Introduction

Lichenization is a successful lifestyle, forming a stable symbiotic association between fungi with cyanobacteria and/or algae. About 13% of the known fungal species form lichens, and these dominate around 7% of the earth’s terrestrial surface [1,2,3]. The origin of lichenization remains controversial. Molecular studies show that lichenization and de-lichenization events occurred independently in different lineages of Ascomycota and Basidiomycota [1,3,4,5,6,7,8,9,10,11,12].

Lecanoromycetes is the largest lichenized lineage in Ascomycota, comprising more than 15,000 species [1,13,14,15]. It currently contains four subclasses: Acarosporomycetidae, Lecanoromycetidae, Ostropomycetidae, and Umbilicariomycetidae [1,16]. Within subclass Ostropomycetidae, Ostropales *sensu lato* exhibits a remarkable transition toward larger, non-lichenized, saprotrophic or biotrophic lineages, including a loss of lichenization within Stictidaceae, making this group the most striking example comprising secondarily delichenized lineages in Lecanoromycetes [1,3,13,17,18].

Ostropales was introduced by Nannfeldt in 1932 to encompass a single family Ostropaceae, which is a younger synonym of Stictidaceae [19]. Various molecular studies have been conducted to resolve the phylogenetic relationships within Ostropales [18,19,20,21,22,23,24,25,26,27,28]. The delimitation of Ostropales has changed over time due to a high level of morphological plasticity [18,19], and the taxonomy of various groups remains unresolved [29]. Ostropales was recently very broadly defined [1] and reduced to a single family, Stictidaceae, whereas related families are now recognized in the separate orders Graphidales, Gyalectales, Odontotrematales, and Thelenellales [13,30]. Stictidaceae includes mostly small, drought-tolerant fungi [31], which have been poorly studied, and their generic delimitation is yet to be resolved [19,31,32]. There are many opportunities for discovering new species, even in well-studied areas [19].

Species of Stictidaceae are mainly saprotrophic and partly lichenized or lichenicolous, and they inhabit mostly bark and rock substrata [32]. Some species show optional lichenization; i.e., the same fungus may be either lichenized when growing on bark or saprotrophic when developing on wood [32]. Many species of Stictidaceae are characterized by ascomata with crystalline excipular incrustations and by long, filiform ascospores [24]. Sherwood [33] provided a detailed monograph of this family with special emphasis on taxa recorded from the USA.

Here, we provide updated multi-gene phylogenetic analyses for Ostropales and related orders focusing on Stictidaceae, thereby describing a newly discovered genus and three new species. Detailed morphological descriptions are provided for the new taxa. In addition, ancestral character state analysis was performed to assess the origin and transition of the various lifestyles occurring in the family.

## 2. Materials and Methods

### 2.1. Phenotypic Analysis

The bark and stem plant materials of newly described taxa were collected from China and Thailand and brought to the laboratory in paper bags. Materials were examined using a Motic SMZ 168 Series microscope. Hand sections of the ascomata were mounted with water, 5% KOH and KI (5% KOH and Lugol’s solution), and examined. Sections of ascomata and other micro-morphological characteristics were photographed using a Nikon ECLIPSE 80i compound microscope fitted with a Canon 550D digital camera. All microscopic measurements refer to dimensions in water and were made with Tarosoft Image Frame Work (0.9.0.7), and images used for figures were processed with Adobe Photoshop CS6 Extended 10.0 software (Adobe Systems, San Jose, CA, USA). The specimens were deposited in the Mae Fah Luang University (MFLU) Herbarium, Chiang Rai, Thailand. Index Fungorum and Faces of Fungi were registered following Index Fungorum [34] and Jayasiri et al. [35].

### 2.2. DNA Extraction, PCR Amplification, and Gene Sequencing

Genomic DNA was extracted directly from the ascomatal tissue and thalli of fungi as outlined by Wanasinghe et al. [36]. An E.Z.N.A.^®^ Forensic DAT (D3591–01, Omega Bio–Tek) DNA extraction kit was used to extract DNA by following the manufacturer’s instructions. DNA samples that were intended for use as a template for PCR were stored at 4 °C for use in regular work, and duplicates were stored at −20 °C for long-term storage. The mitochondrial small subunit spacers (12S, mtSSU), large subunit nuclear rDNA (28S, LSU) and internal transcribed spacers (ITS) were amplified with primer pairs mtSSU1 and mtSSU3R [37], LR0R and LR5 [38], and ITS5 and ITS4 [39]. The PCR amplification for each gene was performed using a final volume of 25 µL, which was comprised of 2.0 µL of DNA template, 1 µL of each forward and reverse primers, 12.5 µL of Taq PCR Super Mix (mixture of Easy Taq TM DNA Polymerase, dNTPs, obtained buffer (Beijing Trans Gen Biotech Co., Chaoyang District, Beijing, China)) and 8.5 µL of sterilized water.

The PCR amplifications were performed following Zoller et al. [37], Vilgalys and Hester [38], and White et al. [39] for the genes mtSSU, LSU, and ITS respectively. Finally, PCR products were examined on 1% agarose electrophoresis gels and stained with ethidium bromide. Purification and DNA sequencing were performed at Shanghai Sangon Biological Engineering Technology & Services Co. (Shanghai, China). The nucleotide sequence data acquired were deposited in GenBank. Alignments and phylogenetic trees were submitted to TreeBASE under submission number 27653.

### 2.3. Phylogenetic Analyses and Species Recognition

The Basic Local Alignment Search Tool (BLAST) search engine of the National Center for Biotechnology Information (NCBI) was used for the preliminary identification of DNA sequences of the new taxa [40]. Sequences of available closely related taxa for Ostropales were retrieved from GenBank (Table 1), including all representatives available of Stictidaceae. Phylogenetic analyses were constructed based on mtSSU, LSU, and ITS sequence data. Outgroup taxa were selected following Lücking [30]. The final combined alignment of Stictidaceae comprised 2530 nucleotide positions and resulted in 107 taxa. We also conducted a multi-marker phylogenetic analysis of Ostropomycetidae to check the placement of Ostropales *sensu stricto* following Kraichak et al. [13] and Lücking [30] for 167 taxa based on mtSSU, LSU, and ITS sequence data. 

Phylogenetic analyses of both individual and combined aligned data were performed under Maximum Likelihood (ML) and Bayesian criteria. The multiple alignments of all consensus sequences, as well as the reference sequences were automatically generated with MAFFT v. 7 [41]. Terminal ends of sequences and ambiguous regions were trimmed manually using BioEdit v. 7.0.5.2 [42] and excluded from the dataset. The phylogenetic web tool “ALTER” [43] was used to convert sequence alignment from FASTA to PHYLIP for RAxML analysis and from FASTA to NEXUS format for Bayesian analysis. The estimated model of ML and Bayesian analyses were performed independently for each locus using MrModeltest v.2.2 [44]. ML was generated using the RAxML-HPC2 on XSEDE (8.2.8) in the CIPRES Science Gateway platform [45] with 1000 separate runs using the GTR+I+G model of evolution. MrBayes v. 3.1.2 was used to perform Bayesian analysis [46]. MCMC was run for 50,000,000 generations, and trees were sampled every 100th generation. The first 10% of trees that represented the burn-in phase were discarded, and only the remaining 90% of trees were used for calculating posterior probabilities (PP) for the majority rule consensus tree. The resulting trees were drawn in FigTree v1.4.0 [47]; then, they were copied to Microsoft PowerPoint 2013 and converted to jpeg files using Adobe Photoshop CS6 Extended 10.0 (Adobe Systems, San Jose, CA, USA).

### 2.4. Ancestral Character State Analyses

We employed ancestral character reconstruction to study the evolutionary history of selected characters [48], specifically lifestyle changes among Ostropales *sensu lato* and possible gains and losses of lichenization. The following lifestyle states were used: lichenized with chlorococcoid algae, lichenized with trentepohlioid algae, non-lichenized saprotrophic and lichenicolous. RASP 3.2.1 (Reconstruct Ancestral State in Phylogenies) was used to conduct ancestral character analysis, using the two approaches, Bayes Traits and Bayesian Binary MCMC [49,50]. Both approaches were performed and visualized using default settings as follows: 1,010,000 iterations for BayesTraits with a burn-in of 10,000, sampling 1000 trees and with 10 ML trees; 50,000 generations for Bayesian Binary MCMC, with 10 chains, a sample frequency of 100, a temperature of 0.1, state frequencies fixed (JC), and among-site rate variation equal.

## 3. Results

### 3.1. Phylogenetic Analyses

**Figure 1:**
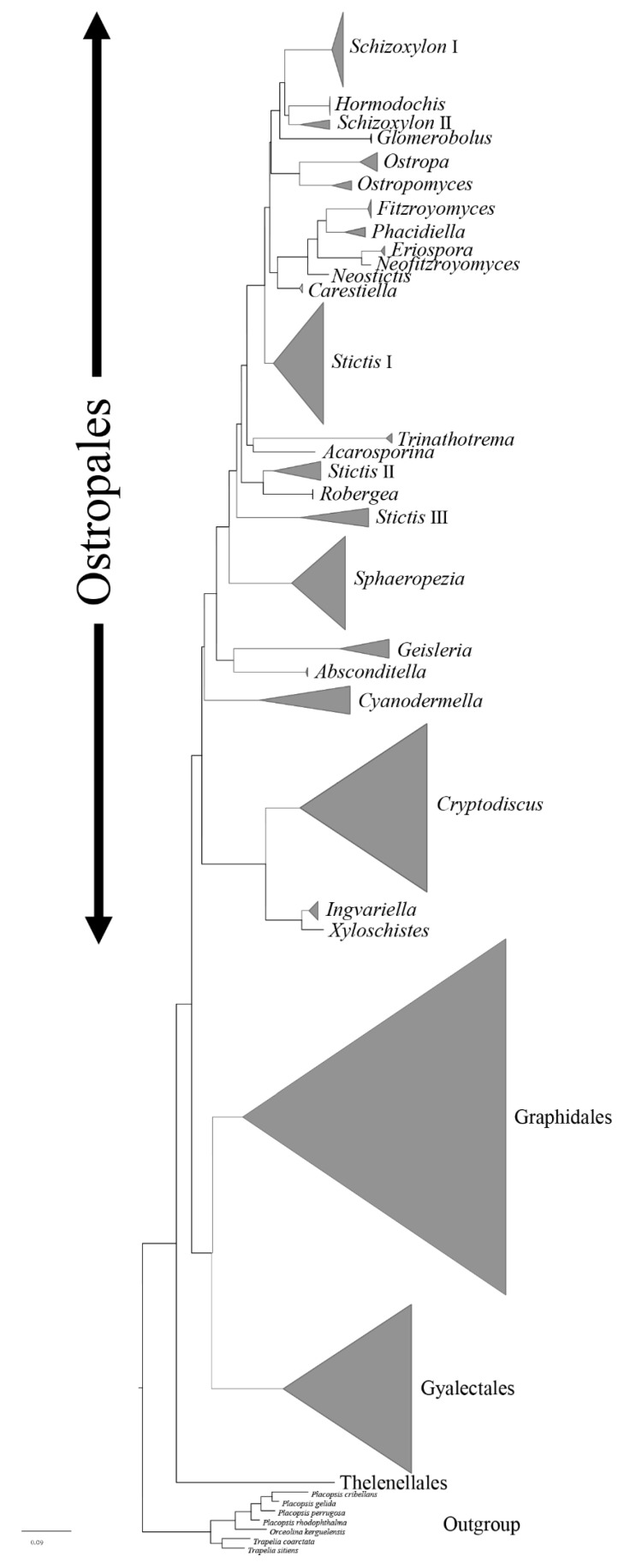
Cartoon tree of major clades for Ostropales *sensu lato* of combined mtSSU, LSU, and ITS partial sequence data based on RAxML tree analysis.

**Figure 2:**
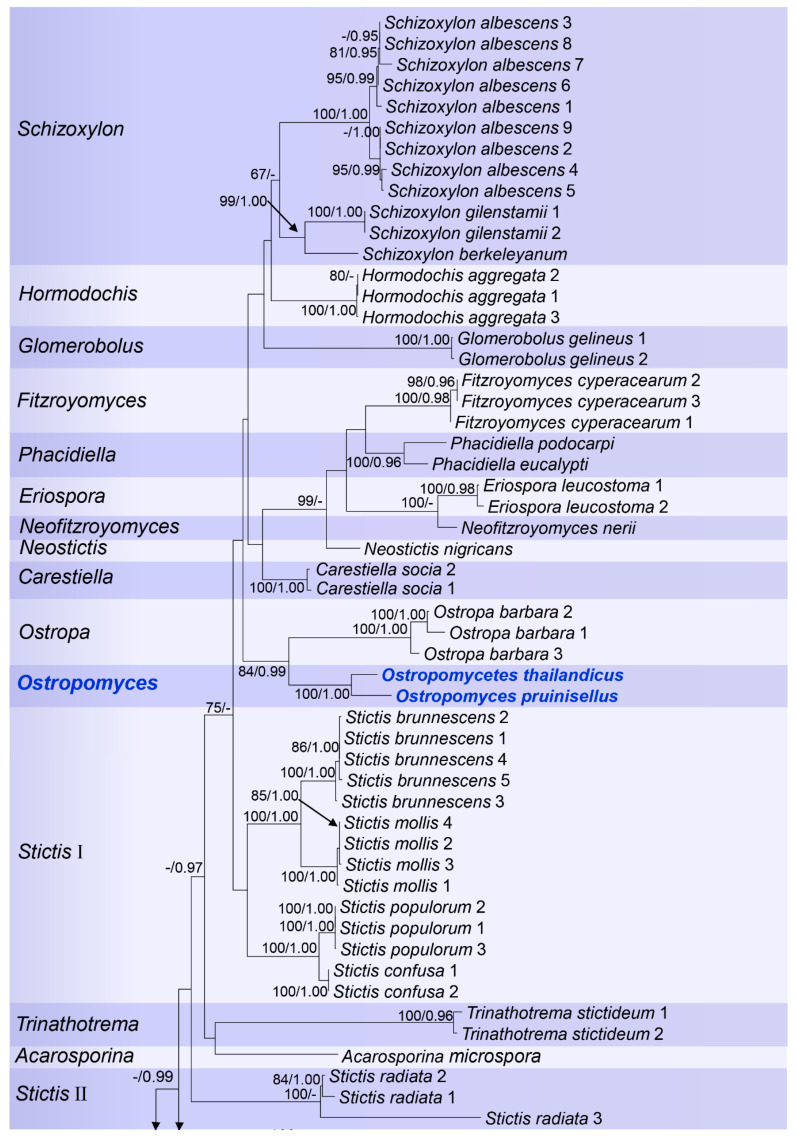

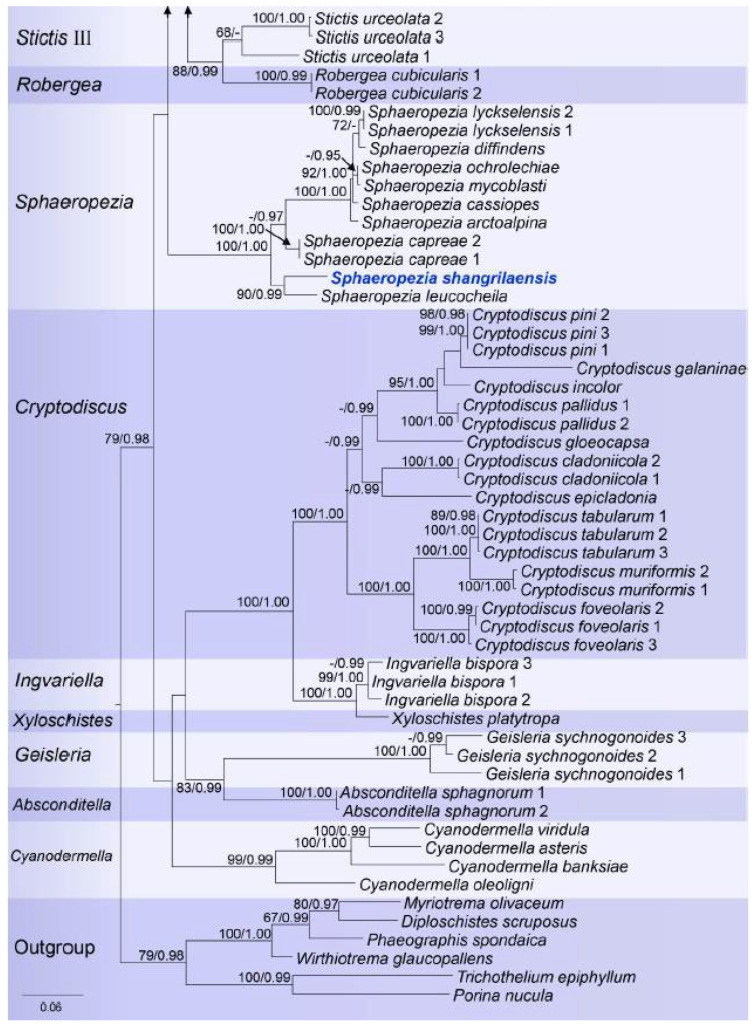
RAxML tree based on analysis of combined mtSSU, LSU, and ITS partial sequence data for Stictidaceae. Bootstrap support values for Maximum Likelihood (ML) equal to or greater than 65%, and Bayesian posterior probabilities (BP) equal to or greater than 0.90 are given as ML/BP above the nodes. The new species and the genus found in this study are displayed in blue bold.

Ostropales *sensu lato* were well recovered including Graphidales, Gyalectales, Ostropales *sensu stricto* (=Stictidaceae), and Thelenellales (Figure 1). No conflict was detected by comparing the significantly supported relationships of the individual topologies of the three markers (mtSSU, LSU, and ITS) that were subsequently concatenated (Supplementary Figures S1–S6). In a second step, we improved the terminal resolution in Stictidaceae by using only closely related lineages as an outgroup (Figure 2). Thereby, Stictidaceae included the following sequenced genera: *Absconditella, Carestiella, Cryptodiscus, Cyanodermella, Eriospora, Fitzroyomyces, Geisleria, Glomerobolus, Hormodochis, Ingvariella, Nanostictis, Neostictis, Neofitzroyomyces, Ostropa*, the new genus *Ostropomyces, Phacidiella, Robergea*, *Schizoxylon, Sphaeropezia, Stictis, Trinathotrema*, and *Xyloschistes*. All genera were resolved as monophyletic except *Stictis* (Figure 2).

The best scoring RAxML tree was selected to represent the relationships among the taxa, with the final ML optimization likelihood value of −29077.976127 (Figure 2). The parameters for the GTR+I+G model of combined mtSSU, LSU, and ITS were as follows: estimated base frequencies A = 0.287442, C = 0.205916, G = 0.252694, T = 0.253948, substitution rates AC = 1.393327, AG = 2.735680, AT = 2.386601, CG = 0.840662, CT = 5.674536 and GT = 1.000000. The ML and Bayesian analyses both resulted in trees with similar topologies. Bayesian posterior probabilities from MCMC were evaluated with a final average standard deviation of split frequencies = 0.005790.

### 3.2. Ancestral Character State Analysis

**Figure 3:**
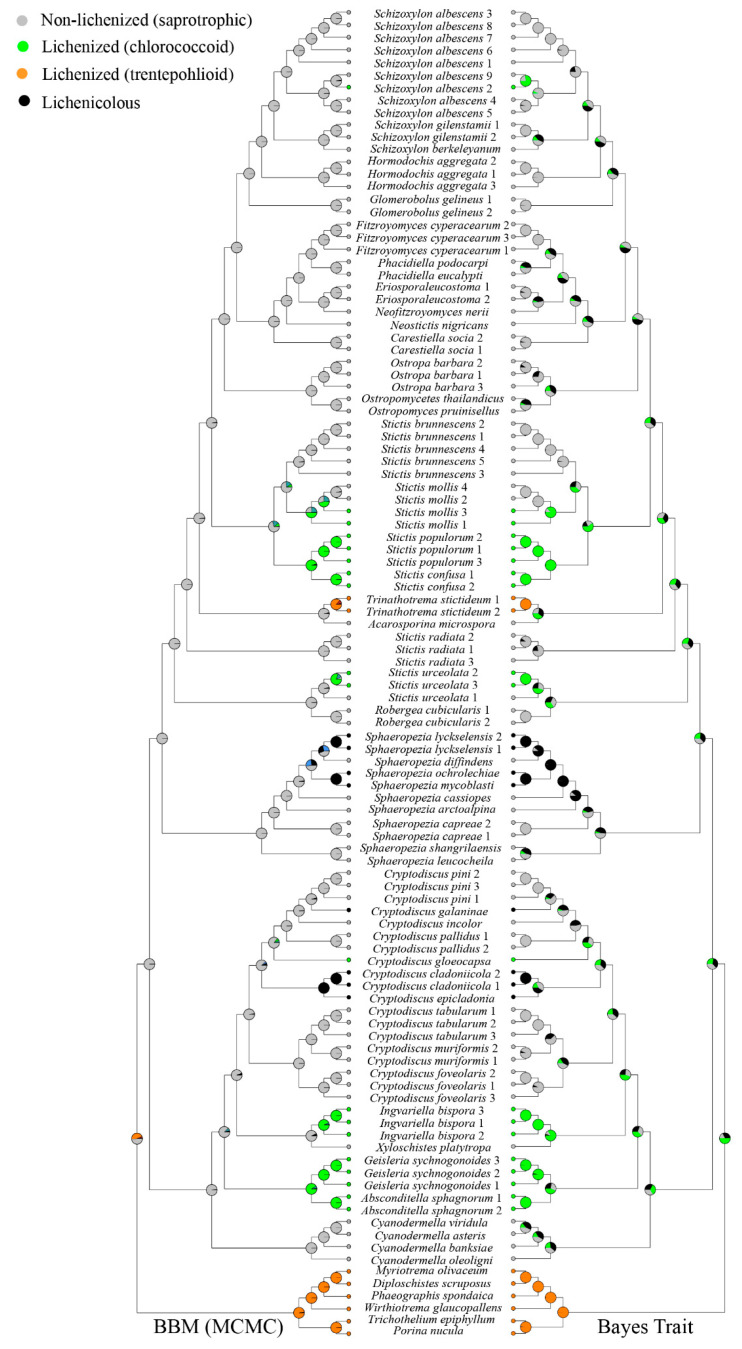
Ancestral character state analysis of Stictidaceae using Bayesian Binary MCMC and Bayes Traits. Color symbols indicate: green = chlorococcoid, orange = trentepohlioid, gray = non-lichenized saprotrophic, black = lichenicolous.

The recently introduced genera, such as *Eriospora*, *Fitzroyomyces, Neofitzroyomyces, Neostictis,* and *Phacidiella*, show saprotrophic lifestyle (Figure 3). *Stictis* was recovered as polyphyletic, with taxa expressing a lichenized or saprotrophic lifestyle or optional lichenization. Most species of *Stictis* show a saprotrophic lifestyle, including the type species of the genus, *Stictis radiata*. *Stictis urceolata,* as well as the lineage formed by *S. populorum* and *S. confusa*, are lichenized with chlorococcoid green algae. *Stictis mollis* is optionally lichenized, with specimens being either saprotrophic (GG2445a, GG2458b) or lichenized (GG2370, GG2440b). Species of *Schizoxylon* also show either a saprotrophic lifestyle or optional lichenization: a lichenized specimen of *S. albescens* (GG2696a) was isolated from the bark of *Populus tremula,* while a saprotrophic specimen (GG236) was isolated from dead twigs and branches of *Populus tremula*. Lichenized and facultatively lichenized Stictidaceae are generally associated with chlorococcoid green algae, except for *Trinathotrema stictideum*, which associates with a trentepohlioid photobiont (Figure 3). The genera *Absconditella* and *Geisleria* form a lichenized lineage, while the lichenized *Ingvariella* is part of a distinct lineage close to the saprotrophic *Xyloschistes platytropa.* Lichenicolous species are nested with saprotrophic species in the genera *Cryptodiscus* and *Sphaeropezia*. Three different lifestyles (lichenized, saprotrophic, and lichenicolous) are present within the genus *Cryptodiscus*. 

Bayesian binary MCMC and Bayes traits analyses give different results regarding the ancestral character analysis. Stictidaceae as a whole was recovered as basally non-lichenized in the Bayesian Binary MCMC tree, suggesting multiple secondary lichenizations of the lichenized lineages within the family. The results for Bayes traits were ambiguous for the basal nodes, not allowing any conclusions about the directionality of lichenization and delichenization.

### 3.3. Taxonomy

**Ostropales** Nannf., Nova Acta Regiae Societatis Scientiarum Upsaliensis, Ser. 4, 8 (2): 68 [51]

Kraichak et al. [13] and Lücking [30] reduced Ostropales *sensu stricto* to the single family Stictidaceae, which is a classification that is followed here.

**Stictidaceae** Fr., Summa vegetabilium Scandinaviae 2: 345, 372 [52]

Syn.: Ostropaceae Rehm (as ‘Ostropeae’), Rabenh. Krypt.-Fl., Edn 2 (Leipzig) 1.3 (lief. 30): 185 (1888) (1896)

Type: *Stictis* Pers., Observationes mycologicae 2: 73 (1800)

Stictidaceae comprises both lichenized and non-lichenized fungi [1,3,18,19,28,31,32,33,53,54,55,56,57,58,59,60,61]. Based on Fries’s classification [62], *Stictis* (including subgen. *Propolis* and subgen. *Xylographa*) and *Cryptomyces* were tentatively included in Stictidaceae. After 1830, the improvement of microscopic-based studies lead to more detailed insight into hymenial configuration. Corda [63] divided immersed, non-stromatic discomycetes into four genera in which he included *Stictis* with unicellular, colorless, and ovoid spores. However, species and generic-level delineation remained uncertain from 1832 to 1932. Fries [52] again assigned *Cryptomyces, Propolis, Xylographa, Naevia,* and *Propolis* to Stictidaceae, ignoring the microscopic classification by Corda. After the inclusion of many genera, Ostropales was erected by Nannfeldt [51] with a single family Ostropaceae. Later, this family was synonymized under Stictidaceae, with the type genus *Stictis* [33,53].

The classification of the family Stictidaceae has changed over time [1,14,15,23,33,52,53,62,63,64,65,66]. Its detailed taxonomy was first studied by Sherwood, focusing on excipular structure, ascospore type, and biology [33,53]. Stictidaceae was traditionally classified as saprotrophic lineage in Ostropales [67]. Gilenstam [68] initially included *Conotrema* as a lichenized genus in the family, whereas currently various lichenized lineages are distinguished, including *Absconditella, Geisleria, Ingvariella*, and *Trinathotrema.* Among these, *Trinathotrema* is the only genus associated with a trentepohlioid photobiont, while other lichenized genera are associated with chlorococcoid photobionts [67,69,70,71]. Winka et al. [72] accepted both lichenized and non-lichenized fungi within this family based on combined multi-gene analysis.

Presently, Stictidaceae comprises 33 genera: *Absconditella*, *Acarosporina*, *Biostictis*, *Carestiella*, *Conotremopsis*, *Cryptodiscus*, *Cyanodermella*, *Delpontia*, *Dendroseptoria*, *Eriospora*, *Fitzroyomyces*, *Geisleria*, *Glomerobolus*, *Hormodochis*, *Ingvariella*, *Karstenia*, *Lillicoa*, *Nanostictis*, *Neostictis*, *Neofitzroyomyces*, *Ostropa*, *Ostropomyces*, *Phacidiella*, *Propoliopsis*, *Robergea*, *Schizoxylon*, *Sphaeropezia*, *Stictis*, *Stictophacidium*, *Thelopsis*, *Topelia*, *Trinathotrema*, and *Xyloschistes* [1,14,15,64]. Generic classification in the family is challenging, given that the convergent evolution of ascoma types is frequent [19] and both apothecoid and perithecoid ascomata have evolved several times in separate lineages [33,73]. However, our updated phylogeny suggests that the only problematic genus at the moment is *Stictis sensu lato*.

***Ostropomyces*** Thiyagaraja, Lücking, Ertz and K.D. Hyde, gen. nov. Index Fungorum number: IF 556555; Faces of Fungi number: FoF 09511 Etymology: name refers to the characteristics similar to *Ostropa*.

Type species: *Ostropomyces pruinosellus* Thiyagaraja, Lücking, Ertz and K.D. Hyde sp. nov.

*Saprobic* on bark, thallus whitish, pruinose. **Sexual morph:** *Ascomata* perithecial, solitary, immersed to erumpent. *Ostiole* distinct. *Exciple* with clear border between outer and inner layer. *Hamathecium* comprising filamentous paraphyses. *Paraphyses* septate, branched, hyaline, filamentous. *Asci* cylindrical, bitunicate. *Ascospores* overlapping uniseriate, hyaline, transversely multi-septate, cells almost of equal size, deeply constricted at the septa of each cell, easily breaking into small septate part-spores. **Asexual morph:** *Pycnidia* erumpent, globose. *Pycnidial wall* in transverse section shows two distinct layers. *Outer layer* hyaline, densely packed. *Inner layer* hyaline, loosely packed, cells elongate in pycnidial neck. *Conidiophores* lining inside and outside of pycnidia wall. *Conidiogenous cells* hyaline. *Conidia* similar in shape to ascospore, filiform, aseptate, hyaline, and guttulate at maturity.

*Notes*: *Ostropomyces* is introduced to accommodate two newly discovered species, *Ostropomyces pruinosellus* and *Ostropomyces thailandicus,* which are collected from tropical forests in Northern Thailand. The new genus is related to *Ostropa*, but both emerge on long stem branches in our phylogenetic analyses (Figure 2). *Ostropomyces* differs from *Ostropa* in the presence of perithecial ascomata, presence of periphysoids, which are present in the inner face of the wall, in the lack of an apical cap in the ascus and four-spored asci. In contrast, *Ostropa* forms orbicular ascomata opening by a transverse slit, periphysoids in the above part, a prominent apical cap in the ascus, and eight-spored or polysporous asci [33]. The new genus formed a distinct clade with high bootstrap support in the multi-gene phylogenetic analyses, whereas its relationship to *Ostropa* was also strongly supported (84%). 

The morphological characteristics would initially suggest that *O. thailandicus* may represent an asexual state of *O. pruinosellus*. However, both lineages formed comparatively long branches in the phylogenetic analysis, indicating that they represent two closely related yet separate species—one known by its sexual morph and the other by its asexual state. Therefore, we introduce *O. thailandicus* and *O. pruinosellus* as new species in *Ostropomyces*. The taxa are characterized by immersed to erumpent fruiting bodies with pseudostromatic masses, orbicular in cross-section, loosely packed hyphae, with numerous periphysoids, numerous, branched, and filiform true paraphyses, long-cylindrical asci without prominent apical cap, four-spored asci, ascospores filiform, colorless, and transversely multi-septate. 

**Figure 4:**
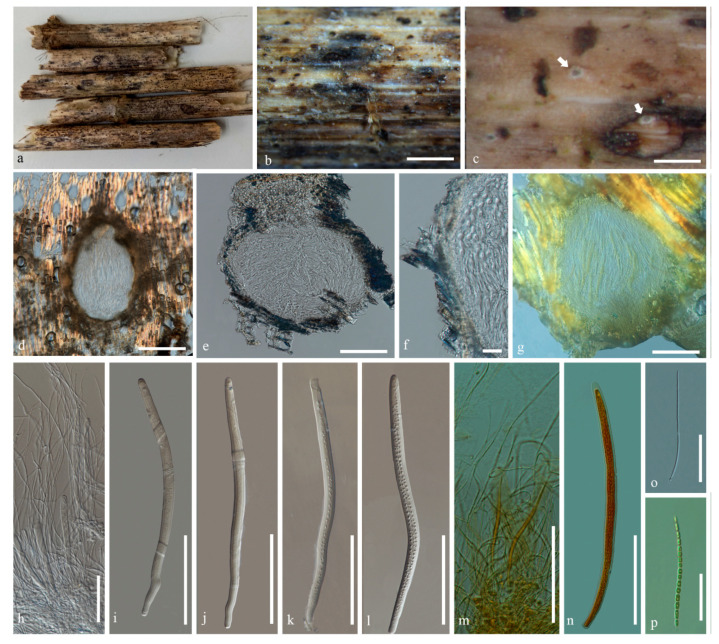
*Ostropomyces pruinosellus* (MFLU 20-0538). (**a**–**c**) Ascomata on substrate. (**d**,**e**) Vertical section through ascoma (in water). (**f**) Vertical section through exciple (in water). (**g**) Vertical section through ascoma (in KI). (**h**) Paraphyses (in water). (**i**–**l**) Asci (in water). (**m**) Paraphyses (in KI). (**n**) Asci (in KI). (**o**) Ascospores (in water). (**p**) Ascospores (in KI). Scale bars b, c = 1000 µm, d, e, g–n = 100 µm, f = 30 µm, o, p = 50 µm.

***Ostropomyces pruinosellus*** Thiyagaraja, Lücking, Ertz and K.D. Hyde, sp. nov. (Figure 4).

Index Fungorum number: IF 556556; Faces of Fungi number: FoF 09512 Etymology: The name refers to the pruinose surface of the substrate where the fungus produces ascomata.

Holotype: MFLU 20-0538

*Saprobic* on unidentified dead stem. Surface of the substrate where the ascomata are formed brownish white, appearing pruinose. *Prothallus* absent. **Sexual morph**: *Ascomata* perithecial, 310–350 μm high, 340–500 μm wide (x = 330 × 420 μm, n = 5), immersed to erumpent, solitary, margin partly protruding beyond the surface layers of stem, not carbonized, color unchanged in KOH, orbicular in cross-section, lined with numerous periphysoids. *Exciple* thickened, outer layer 10–45 μm thick, densely packed, darker than inner layer, inner layer 3–8 μm thick (x = 27.5 × 5.5 μm, n = 10), hyaline, of loosely packed hyphae, with numerous crystalline inclusions and periphysoids extended to the entire inner face of the wall in the 2/3 upper part of the ascomata. *Hamathecium* comprising paraphyses and asci. *Paraphyses* septate, branched, hyaline, 0.5–1.3 μm thick, generally exceeding the length of asci. *Asci* 165–245 × 7–11 μm (x = 205 × 9 μm, n = 40), bitunicate, cylindrical, four-spored, apical wall thickened to 2.2–3.2 μm. *Ascospores* 160–180 × 2–3 μm (x = 170 × 2.5 μm, n = 40), hyaline, transversely multi-septate, each cells almost of equal size, each locus 2–4 μm long, deeply constricted at each septa, easily breaking apart into small, septate, part-spores. **Asexual morph:** Undetermined.

*Spot reactions*: Asci KI-, Ascospores KI-

*Material examined*: Thailand, Mueang Khong, Chiang Dao District, Chiang Mai, N 97°92′86″, E 17°71′45″, 558 m elevation, on unidentified dead stem, 16 February 2019, Vinodhini Thiyagaraja, S1DA (holotype: MFLU 20-0538).

*Notes*: *Ostropomyces pruinosellus* is similar to species in *Ostropa* but differs in the characters listed in the genus discussion. Although the species is saprotrophic and not lichenized, the surface of the substrate where the ascomata emerges has a pruinose appearance, at first glance suggesting the presence of a thallus. However, the apparent thallus is absent. Initially, the ascomata were immersed and became erumpent at maturity.

***Ostropomyces thailandicus*** Thiyagaraja, Lücking, Ertz and K.D. Hyde sp. nov.

Index Fungorum number: IF 556557; Faces of Fungi number: FoF 09513 Etymology: The name refers to the country where the type specimen of the new species was collected.

Holotype: MFLU 20-0539

*Saprobic* on dead stem. Area with pycnidia with a pruinose appearance on the surface. *Prothallus* absent. **Sexual morph**: Undetermined. **Asexual morph**: *Pycnidia* ca 100 μm diam., globose, erumpent, darkening above. *Pycnidial* wall in transverse section composed of two distinct layers. *Outer layer* 19–27 μm wide, hyaline, densely packed, darker than inner layer. *Inner layer* hyaline, loosely packed, 11–23 μm wide. *Conidiophores* reduced to 9–15 μm. *Conidiogenous cells* 9–15 μm, cylindrical, hyaline, lining the inside and outside of the pycnidia wall. *Conidia* 8–13 × 1–3 μm (x = 10.5 × 2 μm, n = 10), filiform, apical proliferation of the conidiogenous cell, aseptate, hyaline.

*Spot reactions*: Conidiophore KI-, Conidia KI-

*Material examined*: Thailand, Mueang Khong, Chiang Dao District, Chiang Mai, N 97°92′86″, E17°71′45″, 558 m elevation, on unidentified dead stem, 16 February 2019, Vinodhini Thiyagaraja, S1D1T2 (holotype: MFLU 20-0539). 

**Figure 5:**
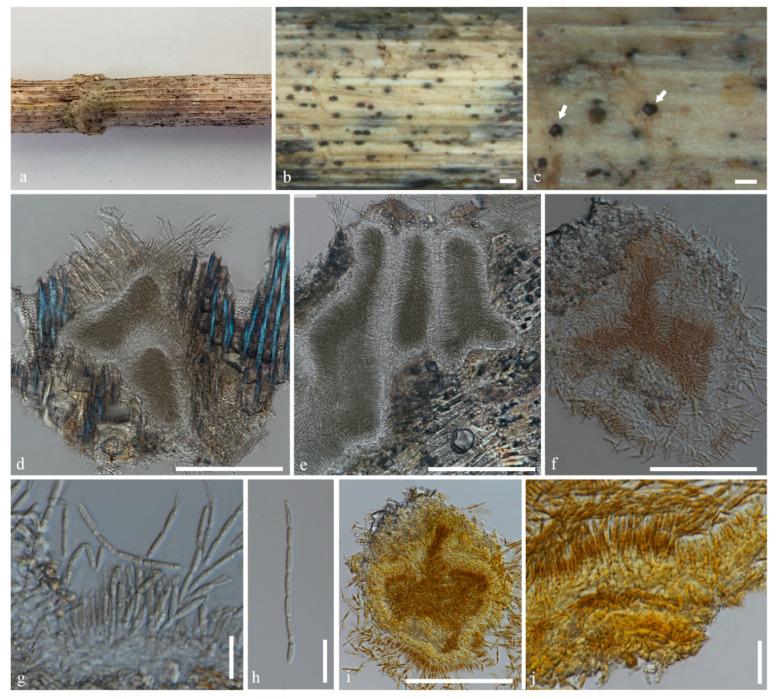
*Ostropomyces thailandicus* (MFLU 20-0539, **holotype**). (**a**–**c**) Pycnidium on substrate. (**d**,**e**) Vertical section through pycnidia (in water). (**f**), Vertical section through pycnidia (in 5% KOH). (**g**) Conidiophores (in water). (**h**) Conidia (in water). (**i**) Vertical section through pycnidia (in KI). (**j**) Conidiophores (in KI). Scale bars b, c = 500 µm, d–f, i = 200 µm, g, h, j = 10 µm.

*Notes*: The new strain was collected from Thailand on the same material from which *Ostropomyces pruinosellus* was isolated (Figure 5). The species are delineated based on DNA sequence data as recommended by Jeewon and Hyde [74]. The phylogenetic tree supported *O. pruinosellus* and *O. thailandicus* as two distinct species, with more than 2% differences in LSU and ITS base pair comparisons. *Ostropomyces thailandicus* formed pycnidial conidiomata, reduced conidiophore into conidiogenous cells, hyaline, and filiform conidia similar to other asexual fungi recorded in Stictidaceae such as *Acarosporina microspora*, *Cyanodermella oleoligni, Stictis radiata,* and *S. urceolata* [28,31,33,68,75].

**Figure 6:**
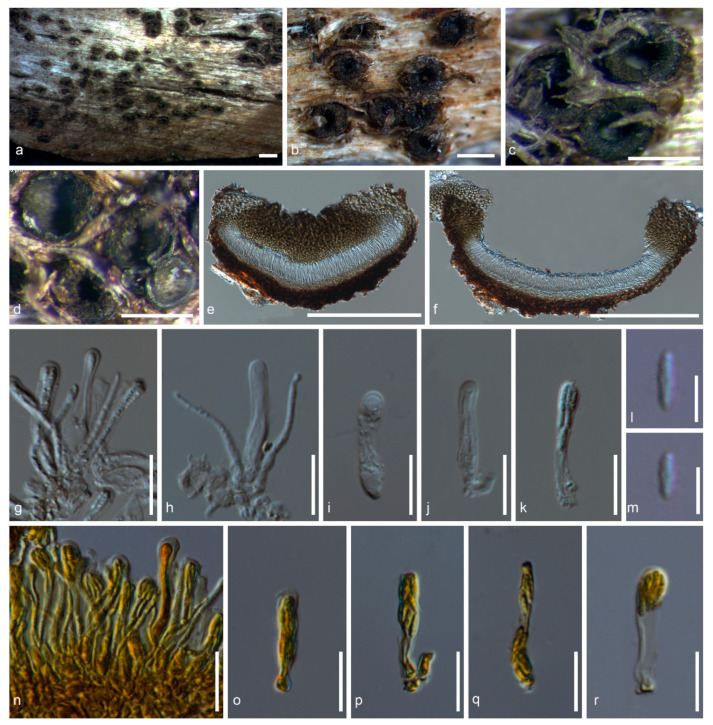
*Sphaeropezia shangrilaensis* (MFLU 20-0537). (**a**–**d**) Ascomata on substrate. (**e**,**f**) Vertical section through an ascoma (in water). (**g**) Paraphyses (in water). (**h**–**k**) Asci (in water). (**l**,**m**) Ascospores (in water). (**n**–**r**) Asci (in KI). Scale bars a = 1000 µm, b–d = 500 µm, e, f = 200 µm, g–k, n–r = 10 µm, l, m = 5 µm.

***Sphaeropeziashangrilaensis*** Thiyagaraja, Lücking, Ertz and K.D. Hyde, sp. nov. (Figure 6) Index Fungorum number: IF 556558; Faces of Fungi number: FoF 09514 Etymology: Refers to the location in China (Shangri-La) where the type specimen was collected.

Holotype: MFLU 20-0537

*Saprobic* on bark. *Thallus* unapparent, surface of the substrate where the ascomata are formed whitish gray, pruinose, crustose, epiphloedal. *Prothallus* absent. *Photobiont* not detected. **Sexual morph**: *Ascomata* apothecial, 345–450 μm diam., black, circular to ellipsoidal, adnate, margin 80–100 μm, slightly erumpent from the thallus, in mature apothecia rolled inward leaving a distinct opening 270–285 μm diam., dark brown, carbonized. *Exciple* 16–38 μm, distinct, dark brown at the base and both sides, light brown in the upper part, 57–87 μm thick. *Hypothecium* 11–21 μm thick, distinct, light brown. *Hymenium* 23–28 μm thick, hyaline. *Epihymenium* 3–7 μm thick, hyaline. *Paraphyses* 1–2.4 μm wide, hyaline, densely arranged. *Asci* 21–24 × 4–6 μm (x = 22.5 × 5 μm, n = 40), hyaline, clavate to obovoid, eight-spored but sometimes four-spored when immature, unitunicate, multiseriate, tip blunted, not narrowing towards the apex, tholus thickened, lacking an apical cap, with poorly developed stipe. *Ascospores* 4–6 × 0.7–1.0 μm (x = 5 × 0.85 μm, n = 40), hyaline, smooth–walled, fusoid to obovoid, (0–)1-septate. **Asexual morph**: Undetermined

*Spot reactions*: Ascomatal gel I-, KI-. Hymenium I-, KI-. Asci I-, KI-. Ascospores I-, KI-

*Material examined*: China, Yunnan Province, Shangri La, N 27°55′05.8″, E 99°36′33.4″, 3964 m elevation, on unidentified dead bark, 14 September 2018, Vinodhini Thiyagaraja, D6S51 (holotype: MFLU 20-0537)

*Notes*: *Sphaeropezia* was resurrected by Baloch et al. [18] and comprises 22 species with *S. alpina* as the type [34]. *Sphaeropezia* was originally introduced by Saccardo [76] and associated with *Odontotrema* with the special adaptation to a foliicolous growth and was assigned to Odontotremataceae due to shared morphological characteristics [18]. However, *Sphaeropezia* was placed in Stictidaceae based on molecular data and some *Bryodiscus* species, which had been recorded as parasites on mosses, were also transferred to *Sphaeropezia* [18]. 

Species of this genus are characterized by dark-walled, deeply urceolate apothecia, mostly erumpent at maturity, living as saprobes on wood or herbaceous material, or as putative parasites of bryophytes or lichens. They are distributed mainly in northern temperate regions [18]. The new taxon was collected from the sub-tropical region of southwestern of Shangri la, China, which is one of the world’s biodiversity hotspots [77]. *Sphaeropezia shangrilaensis* clustered together with *S*. *leucocheila* and formed a clade with *S*. *capreae* with high statistical support in the multi-gene phylogenetic analyses. The new taxon differs from other *Sphaeropezia* species in the larger pore opening in ascomata and the smaller asci (Figure 6; Table 2). 

Specifically, *Sphaeropezia shangrilaensis* differs from *S. capreae* in the position of the ascomata (superficial vs. fully erumpent), the larger ascomatal pore opening (273–283 μm vs. (60–)100–150(–200)) μm, the smaller asci (21–24 × 4–5 μm vs. 55–65 × 8–10 μm), the shape of ascospores (bacilliform vs. fusoid to obvoid), and the number of ascospores per asci (4 to 8 vs. polyspored). *Sphaeropezia shangrilaensis* also differs from *S. leucocheila* in the shape of the ascomata (roundish vs. globose), the larger pore opening (273–283 μm vs. 80 μm), the smaller asci (21–24 × 4–5 μm vs. 50–55 × 6–8 μm), and the size of the ascospores (4–6 × 0.7–1.0 μm vs. 8–11.5 × 2–3 μm) [78]. *Sphaeropezia shangrilaensis* is only known from China while *S. capreae* and *S. leucocheila* were recorded from Sweden and New Zealand, respectively [18,78].

## 4. Discussion

Molecular phylogenetic studies show that lichenization occurred several times independently in both Ascomycota and Basidiomycota [4,5,6,7,9,10]. Baloch et al. [19] concluded that independent saprotrophic lineages in Ostropales *sensu lato* resulted from multiple losses of lichenization. Lutzoni et al. [20] also stated that non-lichenized ostropalean species were derived from a lichenized ancestor. These findings have been confirmed by other recent studies [3,12], whereas others indicated a deeper loss of lichenization in the clade leading to Stictidaceae (Figure 3). The latter was in part also supported by our own analysis using Bayesian MCMC, suggesting multiple independent relichenization in the family, although the results from Bayes traits were ambiguous. 

One lichenized genus that we did not include in our analysis of Stictidaceae was *Topelia*. The genus comprises eleven species, but molecular data are lacking except for the type species. In our multi-gene phylogenetic analyses, *T. rosea* formed a comparatively long branch, and its position relative to Stictidaceae was unstable. Stictidaceae is not the only family in Ostropales *sensu lato* showing close relationships of lichenized and saprotrophic lineages. The predominantly lichenized family Graphidaceae now also contains the saprotrophic species *Furcaspora eucalypti* and *Rubikia evansii*, apparently derived from a lichenized ancestor [80], and *Agyrium* in Pertusariales was also derived through delichenization [23]. Stictidaceae itself contains a wide diversity of lifestyles, which may vary not only at genus but also at the species level [32,60,61]. The biology of some taxa (e.g., *Lillicoa palicprea* and *Delpontia*) remains unresolved [33]. 

Apart from lichenized and saprotrophic lineages, the lichenicolous lifestyle appeared multiple times independently within Stictidaceae, as shown previously by Pino-Bodas et al. [81]. Aptroot [82] and Cáceres et al. [80] suggested that delichenization can lead to both lichenicolous and saprotrophic lifestyles, which is supported by our analysis. Aptroot [82] stated that relichenization is a rare case, often resulting in loosely associated lichenized forms. In this respect, optionally lichenized fungi such as *Stictis mollis* and *Schizoxylon albescens* are of interest, as they seem to be derived from non-lichenized ancestors. Several species of the saprotrophic genus *Acarosporina* also have been recorded as parasitic, causing cankers on *Quercus* and *Fagus* in eastern North America [33]. *Cyanodermella* comprises saprotrophic fungi [83], and at least one species, *C. asteris*, has been recorded as endophytic. Several species of the lichenized genus *Absconditella* have been recorded as pathogens on bryophytes [84]. Thus, lifestyle switches may drive evolution in Stictidaceae and potentially drive speciation, but this needs to be tested with a much broader sampling, especially of *Stictis sensu lato.* Lifestyle switches are overall unusually frequent in Ostropales *sensu lato*, showing the evolutionary plasticity of this enigmatic group [26,85,86,87]. More detailed molecular studies and increased taxon sampling are also needed to resolve generic and species-level limits in the family [31]. Surprisingly, our phylogeny suggests that the only problematic genus at this point is the polyphyletic *Stictis sensu lato*. 

## Figures and Tables

**Table 1:** Taxa used in this study for the analyses of combined mitochondrial small subunit spacers (mtSSU), large subunit nuclear rDNA (LSU), and internal transcribed spacers (ITS) sequence data and their GenBank accession numbers. The newly generated sequences are indicated in boldface.

		GenBank Accession Numbers		
Species	Strains	mtSSU	LSU	ITS
*Absconditella sphagnorum* 1	T. Laukka 52 (TUR)	EU940247	EU940095	–
*Absconditella sphagnorum* 2	17 Feb 02 Palice (HB Palice)	AY300872	AY300824	–
*Acarosporina microspora*	AFTOL-ID 78	AY584612	AY584643	DQ782834
*Carestiella socia* 1	GG2410	AY661677	AY661687	AY661687
*Carestiella socia* 2	GG2437a	AY661678	AY661682	AY661682
*Cryptodiscus cladoniicola* 1	RP160	KY661675	KY661653	KY661620
*Cryptodiscus cladoniicola* 2	RP159	KY661674	KY661652	KY661619
*Cryptodiscus epicladonia*	RP208	KY661680	–	KY661628
*Cryptodiscus foveolaris* 1	EB155	FJ904695	–	FJ904673
*Cryptodiscus foveolaris* 2	EB86	FJ904692	–	FJ904670
*Cryptodiscus foveolaris* 3	EB147	FJ904694	–	FJ904672
*Cryptodiscus galaninae*	RP314	–	–	KY661636
*Cryptodiscus gloeocapsa*	EB93	FJ904696	–	FJ904674
*Cryptodiscus incolor*	EB164	FJ904697	–	FJ904675
*Cryptodiscus muriformis* 1	UPS F-647154	MG281972	MG281962	MG281962
*Cryptodiscus muriformis* 2	H.B. 6773	MG281973	MG281963	MG281963
*Cryptodiscus pallidus* 1	EB60	FJ904700	FJ904678	FJ904678
*Cryptodiscus pallidus* 2	EB173	FJ904702	FJ904680	FJ904680
*Cryptodiscus pini* 1	EB82	FJ904704	FJ904682	FJ904682
*Cryptodiscus pini* 2	EB178	FJ904705	FJ904683	FJ904683
*Cryptodiscus pini* 3	EB181	FJ904706	FJ904684	FJ904684
*Cryptodiscus tabularum* 1	CO205	FJ904712	FJ904690	FJ904690
*Cryptodiscus tabularum* 2	EB169	FJ904711	FJ904689	FJ904689
*Cryptodiscus tabularum* 3	EB77	FJ904709	FJ904687	FJ904687
*Cyanodermella asteris*	03HOR06-2-4	–	KT758843	KT758843
*Cyanodermella banksiae*	CPC:32105	–	NG_064548	NR_159835
*Cyanodermella oleoligni*	DTO 301-G1	KX999144	KX950461	KX950434
*Cyanodermella viridula*	EB146	–	MG281964	MG281964
*Diploschistes scruposus*	SFB 95	KC167052	–	KC167001
*Eriospora leucostoma* 1	CPC:35594	–	MT223890	MT223795
*Eriospora leucostoma* 2	CPC:35598	–	MT223891	MT223796
*Fitzroyomyces cyperacearum* 1	CPC:32209	–	NG_058513	NR_156387
*Fitzroyomyces cyperacearum* 2	MFLU 18-0695b	–	MK499361	MK499349
*Fitzroyomyces cyperacearum* 3	MFLU 18-0695a	–	MK499363	–
*Geisleria sychnogonoides* 1	Caceres & Aptroot 13560 (ABL)	KC689751	KC689752	–
*Geisleria sychnogonoides* 2	GESY7510	KF220306	KF220304	–
*Geisleria sychnogonoides* 3	GESY7509	KF220305	–	–
*Glomerobolus gelineus* 1	AFTOL-ID 1349	DQ247784	DQ247803	DQ247782
*Glomerobolus gelineus* 2	JK 5584C	DQ247783	DQ247798	–
*Hormodochis aggregata* 1	CBS:145904	–	–	NR_166307
*Hormodochis aggregate* 2	CPC:37499	–	MN317288	MN313807
*Hormodochis aggregata* 3	CPC:35475	–	MN317287	MN313806
*Ingvariella bispora* 1	DUKE 1444446	HQ659175	–	–
*Ingvariella bispora* 2	MALich 15288	HQ659173	HQ659184	–
*Ingvariella bispora* 3	BCNLich 17183	HQ659174	HQ659185	–
*Myriotrema olivaceum*	Kalb 39107	KJ435181	KJ435111	–
*Neofitzroyomyces nerii*	CBS:145088	–	MK047504	MK047454
*Neostictis nigricans*	MFLU 18-1380	–	MT214610	MT310654
*Ostropa barbara* 1	S F302817	MG281974	MG281965	MG281965
*Ostropa barbara* 2	EB85	HM244752	HM244773	HM244773
*Ostropa barbara* 3	G. M. 2015-04-28.1	–	KY608095	KY608095
***Ostropomyces pruinosellus***	**MFLU 20-0538**	**MW400963**	**MW400966**	**MW400964**
***Ostropomyces thailandicus***	**MFLU 20-0539**	**–**	**MW397060**	**MW400967**
*Phacidiella eucalypti*	CBS 120255	–	MT373344	MT373361
*Phacidiella podocarpi*	CBS 138904	–	NG_058118	NR_137934
*Phaeographis spondaica*	Lumbsch 19633	JX421280	–	–
*Porina nucula*	Lücking 17007-c	KJ449310	–	–
*Robergea cubicularis* 1	G.M. 2013-05-09.1	–	KY611899	KY611899
*Robergea cubicularis* 2	G.M. 2017-10-12.1	–	MN833317	MN833317
*Schizoxylon albescens* 1	GG236	AY661680	AY661689	AY661689
*Schizoxylon albescens* 2	GG2696a	DQ401142	DQ401144	DQ401144
*Schizoxylon albescens* 3	Wedin 8365 (S)	–	–	HQ287353
*Schizoxylon albescens* 4	Wedin 8364 (S)	–	–	HQ287352
*Schizoxylon albescens* 5	Wedin 8356 b (S)	–	–	HQ287350
*Schizoxylon albescens* 6	Wedin 8359 (S)	–	–	HQ287351
*Schizoxylon albescens* 7	Wedin 8327 (S)	–	–	HQ287349
*Schizoxylon albescens* 8	Wedin 8324 (S)	–	–	HQ287348
*Schizoxylon albescens* 9	Wedin 8254 (S)	–	–	HQ287347
*Schizoxylon berkeleyanum*	F209682	MG281975	MG281966	MG281966
*Schizoxylon gilenstamii* 1	MW9490	MG281977	MG281968	MG281968
*Schizoxylon gilenstamii* 2	MW9496	MG281978	MG281969	MG281969
*Sphaeropezia arctoalpina*	Baloch SW057	HM244736	HM244760	–
*Sphaeropezia capreae* 1	GG2560	AY661674	AY661684	–
*Sphaeropezia capreae* 2	UPS (Gilenstam 2633a)	HM244751	HM244772	–
*Sphaeropezia cassiopes*	Baloch s.n. (S)	HM244746	–	–
*Sphaeropezia diffindens*	Baloch SW020 (S)	HM244747	–	–
*Sphaeropezia leucocheila*	PDD 98299	MK547101	MK547099	MK547090
*Sphaeropezia lyckselensis* 1	Gilenstam 2651 (S)	JX266156	JX266158	–
*Sphaeropezia lyckselensis* 2	Gilenstam 2659	HM244750	HM244771	–
*Sphaeropezia mycoblasti*	Wedin 8509 & Westberg (S)	JX266157	JX266159	–
*Sphaeropezia ochrolechiae*	Wedin 6729 (UPS)	–	JX266160	–
***Sphaeropezia shangrilaensis***	**MFLU 20-0537**	**MW400962**	**MW400965**	**MW400955**
*Stictis brunnescens* 1	EB84	MG281979	–	–
*Stictis brunnescens* 2	Gilenstam 2359 (UPS)	AY661679	–	AY661688
*Stictis brunnescens* 3	SFB1100	MG281981	–	MG281970
*Stictis brunnescens* 4	MW8571	MG281980	–	–
*Stictis brunnescens* 5	SFB1105	MG281982	–	MG281971
*Stictis confusa* 1	Wedin 7070 (UPS)	DQ401141	–	DQ401143
*Stictis confusa* 2	AN3222	AY527365	–	AY527336
*Stictis mollis* 1	GG2440b	AY527342	–	AY527313
*Stictis mollis* 2	GG2445a	AY527347	–	AY527318
*Stictis mollis* 3	GG2370	AY527339	–	AY527310
*Stictis mollis* 4	GG2458b	AY527345	–	AY527316
*Stictis populorum* 1	GG2618	AY527360	–	AY527331
*Stictis populorum* 2	GG2610a	AY527356	–	AY527327
*Stictis populorum* 3	MW7301	AY527363	–	AY527334
*Stictis radiata* 1	MW6493	AY527338	–	AY527309
*Stictis radiata* 2	GG2449a	AY340532	–	AY527308
*Stictis radiata* 3	AFTOL-ID 398	AY584727	–	DQ782846
*Stictis urceolata* 1	MFLU 19–2695	–	MN989186	–
*Stictis urceolata* 2	LT21500	AY661676	AY661686	AY661686
*Stictis urceolata* 3	AFTOL-ID 96	–	–	HQ650601
*Trichothelium epiphyllum*	Baloch CR-127	AY648901	–	–
*Trinathotrema stictideum* 1	F:Luecking 17541b	GU380288	–	–
*Trinathotrema stictideum* 2	F:Luecking 28093	GU380287	–	–
*Wirthiotrema glaucopallens*	DNA1336	JF828972	–	–
*Xyloschistes platytropa*	H:Bjork 05-242	KJ766517	KJ766680	–

**Table 2:** Synopsis of recorded *Sphaeropezia* species.

Species Name	Position of Ascoma	Shape of Ascoma	Size of Ascoma (μm)	Size of Ascoma Pore Opening (μm)	Size of Asci (μm)	Spore Size (μm)	Ascospore Shape	Number of Septate	Known Distribution	Reference
*Sphaeropezia santessonii*	Immersed, partly erumpent, finally sessile	-	(225–) 280–380 (–440)	(20–) 55–125 (–190)	40–50 (–55) × 8–13	(12·5–) 15·4–20·4 (–23·5) × (3–) 3·6–4·6(–5)	Fusiform, often asymmetrical	trans-septate (3–) 6–8 (–9) to submuriform	Russian Arctic, Iceland and Peru, widespread and common in Arctic regions	[79]
*S. bryoriae*	Superficial	Roundish to subspherical	(275–) 310–410 (–440)	(0–) 10–70 (–120)	40–60 × 5–6	(7·4–) 7·6–8·8 (–9·2) × (2·8–)3· 1–3·5(–4·0)	Ellipsoid	1-septate (exceptionally 2-septate)	USA (Washington)	[79]
*S. capreae*	Fully erumpent -		(280–) 350–450	(60–) 100–150 (–200)	55–65 × 8–10	(4–)5–7(–8) × 1–1.3(–1.5)	Bacilliform	-	Sweden	[18]
*S. leucocheila*	Superficial	Globose-urceolate	Up to 300	80	50–55 × 6–8	8–11.5 × 2–3	Oblong-elliptic	(0–) 1-septate	New Zealand	[78]
*S. lyckselensis*	Erumpent	-	(175–) 250–350 (–425)	(25–) 40–75 (–125)	35–60 × 5–6.5	-	Cylindrical oblong	3-septate	Northern Sweden	[18]
*S. melaneliae*	Immersed	Roundish	170–350	0–20	60–85 × 6·5–8·5	(12–)12·8–14·4 (–15·5) × (5·4–) 5·5–6·1 (–6·3)	Ellipsoid	(1–)3-septate, exceptionally with one longitudinal septum	Sweden and Alaska	[79]
*S. mycoblasti*	Erumpent	-	(140–) 190–280 (–320)	(0–) 20–50 (–70)	50–70 × 7–9	(12.3–) 14.0–15.9 (–17) × (4.0–) 4.7–5.3 (–5.7)	Ellipsoid to narrowly ellipsoid	3-septate, (exceptionally 4-septate)	USA (Oregon) and northern Sweden	[18,70]
*S. ochrolechiae*	Immersed and become erumpent	-	(180–) 230–330 (–400)	(0–) 5–50 (–150)	50–75 × 9–14	(10·8–) 12·1–14·4 (–16·0) × (4·3–) 4·8–5·5 (–6)	Ellipsoid to narrowly ellipsoid	3-septate	Norway, Sweden and the USA (Alaska)	[79]
*S. pertusariae*	Immersed to erumpent	-	(140–) 170–260 (–310)	(20–) 40–110 (–150)	-	(11·5–)12·5–15·4 (–16·0) × (4·5–) 4·7 –5·5 (–6·0)	Ellipsoid	1–3-septate	Great Britain (Scotland)	[79]
*S. rhizocarpicola*	Immersed and occasionally erumpent	Roundish	(140–) 155–245 (–300)	(30–) 30–60 (–70)	50–70 × 6·5–13	(8·0–)9·3–11·1 (–13·5) × (4·5–) 4·8–5·6(–6·5)	-	(1–)3-septate	Russia, Kola and Peninsula	[79]
*S. santessonii*	Immersed, -finally sessile partly erumpent	-	(225–) 280–380 (–440)	(20–) 55–125 (–190)	40–50 (–55) × 8–13	(12·5–)15·4–20·4 (–23·5) × (3–) 3· 6–4·6 (–5)	Fusiform, often asymmetrical	Trans-septate (3–) 6–8 (–9) to submuriform	Widespread and common in Arctic regions	[79]
*S. sipei*	Immersed, soon erumpent	Sub-spherical	(350–) 360–480 (–590)	(0–) 0–40 (–105)	55–65 × 5–7	(11·0–)12·2–13·8 (–14·5) × (4·2–) 4·5–5·0 (–5·0)	Ellipsoid to narrowly ellipsoid	3-septate	USA (Oregon) and Canada (British Columbia)	[79]
*S. thamnoliae*	Immersed and occasionally sessile	Roundish or slightly ellipsoid	(140–) 150–200 (–290)	(0–) 20–60 (–85)	30–45 × 7–10	(9·0–)11·0–14·9 (–18·0) × (2·5–) 2·5–3·2 (–3·5)	Fusiform	1(–2)-septate	Russian and Swedish Arctic	[79]
*S. shangrilaensis*	Slightly erumpent to superficial	Roundish	345–446	273–283	21–24 × 4–5.5	4–6 × 0.7–1.0	Fusoid to obvoid	(0–) 1-septate	China	This study

## Data Availability

Not applicable.

## Supplementary Materials

The following are available online at https://www.mdpi.com/2309-608X/7/2/105/s1. Figure S1. Best-scoring RAxML tree reconstructed based on analysis of a single dataset of mtSSU sequence data. Bootstrap support values for ML equal to or greater than 65% is defined above the nodes. Figure S2. Best-scoring RAxML tree reconstructed based on analysis of a single dataset of LSU sequence data. Bootstrap support values for ML equal to or greater than 65% is defined above the nodes. Figure S3. Best-scoring RAxML tree reconstructed based on analysis of a single dataset of ITS sequence data. Bootstrap support values for ML equal to or greater than 65% is defined above the nodes. Figure S4. Best-scoring RAxML tree reconstructed based on analysis of a single dataset of mtSSU sequence data. Bootstrap support values for BP equal to or greater than 0.90 is defined above the nodes. Figure S5. Best-scoring RAxML tree reconstructed based on analysis of a single dataset of LSU sequence data. Bootstrap support values for BP equal to or greater than 0.90 is defined above the nodes. Figure S6. Best-scoring RAxML tree reconstructed based on analysis of a single dataset of ITS sequence data. Bootstrap support values for BP equal to or greater than 0.90 is defined above the nodes.
