# Supplementary material for: The Evolution of Life Modes in Stictidaceae, with Three Novel Taxa
Source: J Fungi (Basel). 2021 Feb 2;7(2):105. doi: 10.3390/jof7020105 (PMC7913076; doi:10.3390/jof7020105)

Supplementary Materials:

Figure S1. Best-scoring RAxML tree reconstructed based on analysis of a single dataset of mtSSU sequence data. Bootstrap support values for ML equal to or greater than 65% is defined above the nodes.

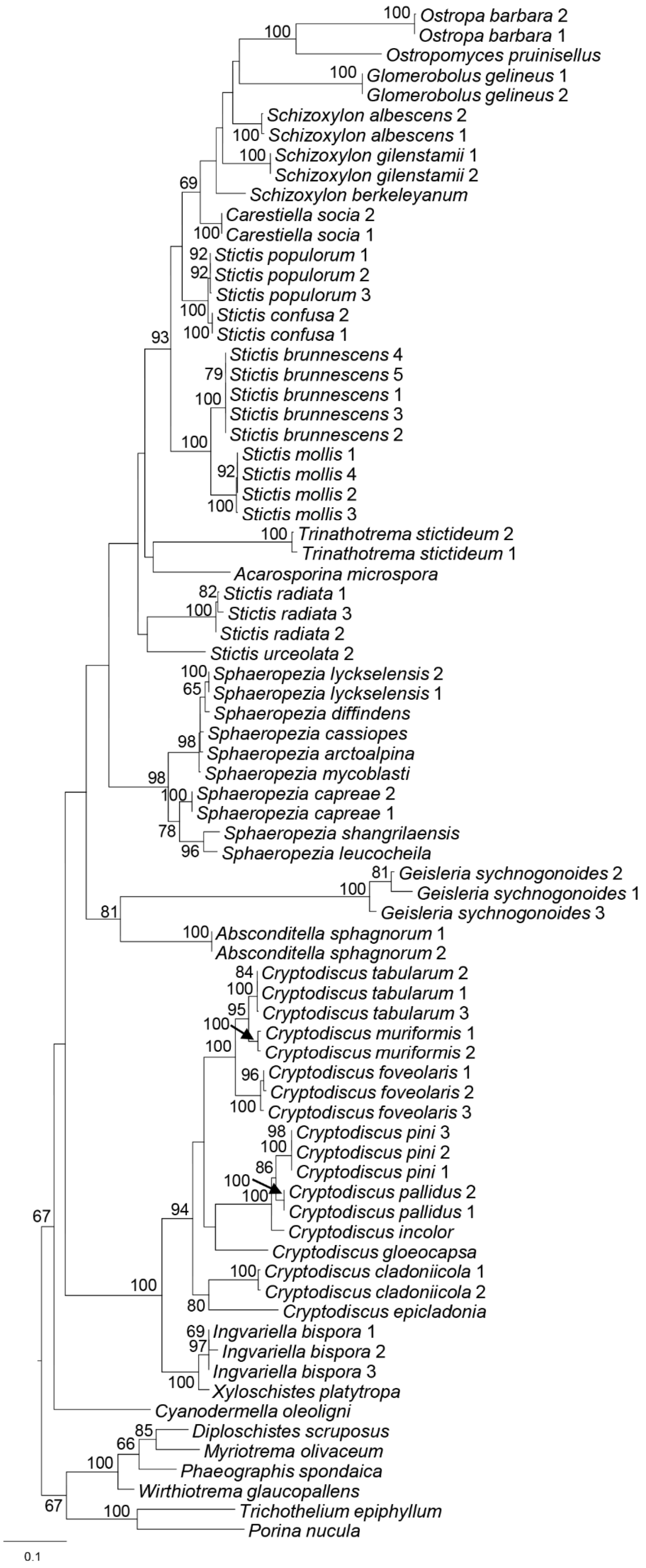

Figure S2 Best-scoring RAxML tree reconstructed based on analysis of a single dataset of LSU sequence data. Bootstrap support values for ML equal to or greater than 65% is defined above the nodes.

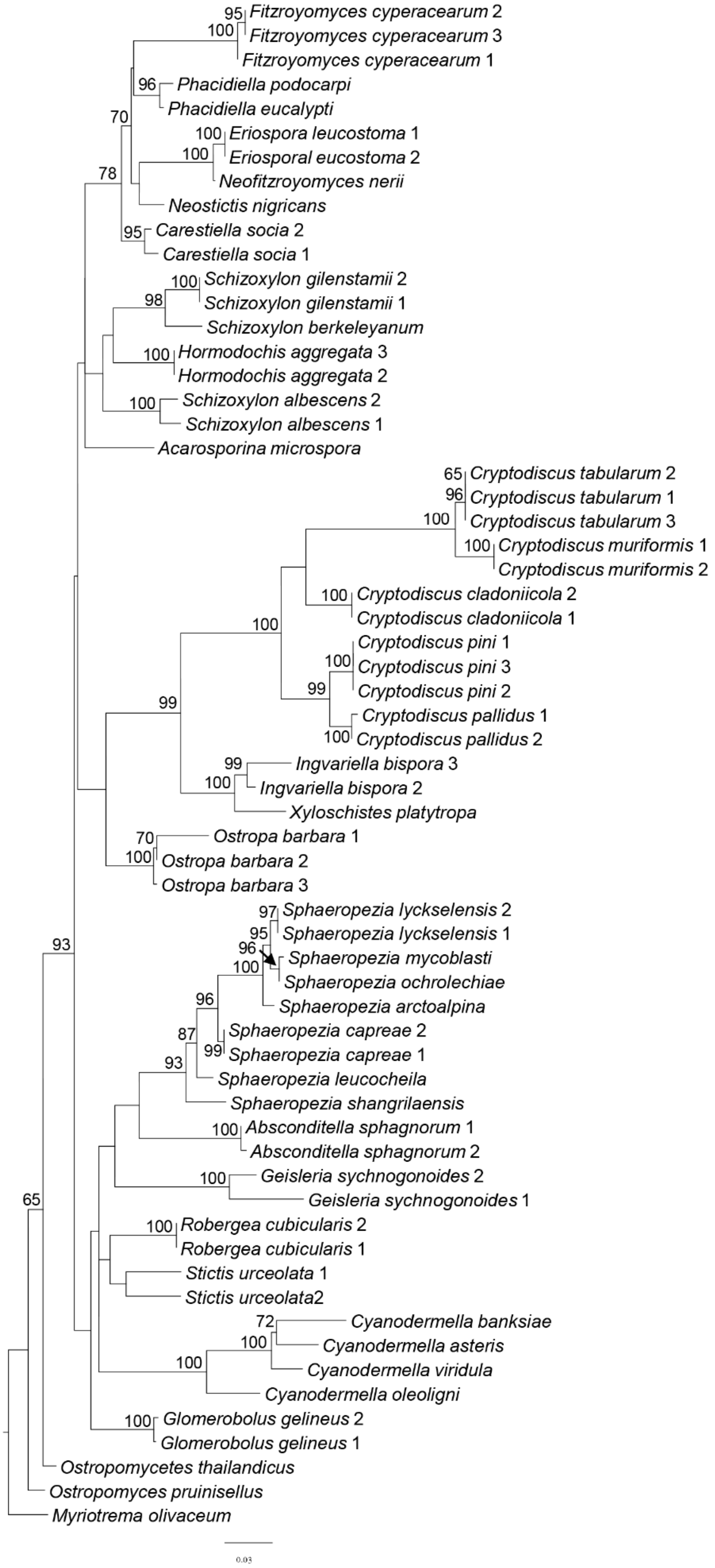

Figure S3 Best-scoring RAxML tree reconstructed based on analysis of a single dataset of ITS sequence data. Bootstrap support values for ML equal to or greater than 65% is defined above the nodes.

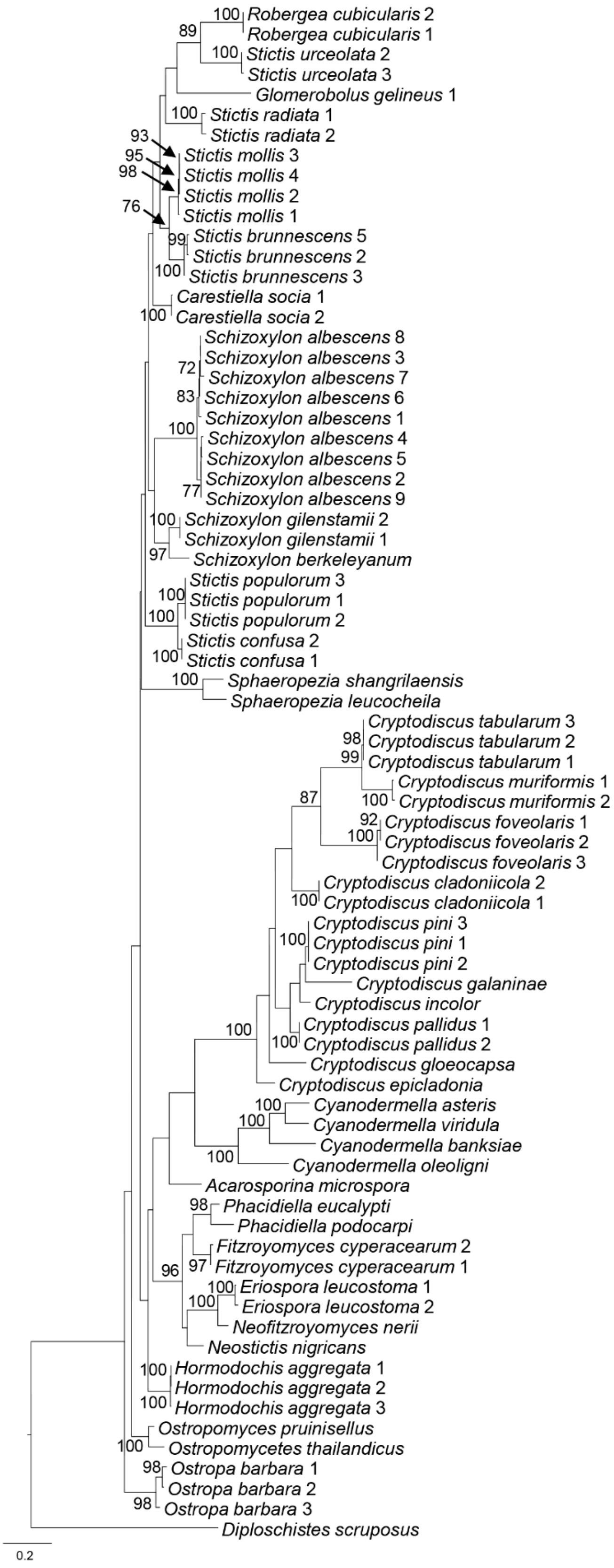

Figure S4 Best-scoring RAxML tree reconstructed based on analysis of a single dataset of mtSSU sequence data. Bootstrap support values for BP equal to or greater than 0.90 is defined above the nodes.

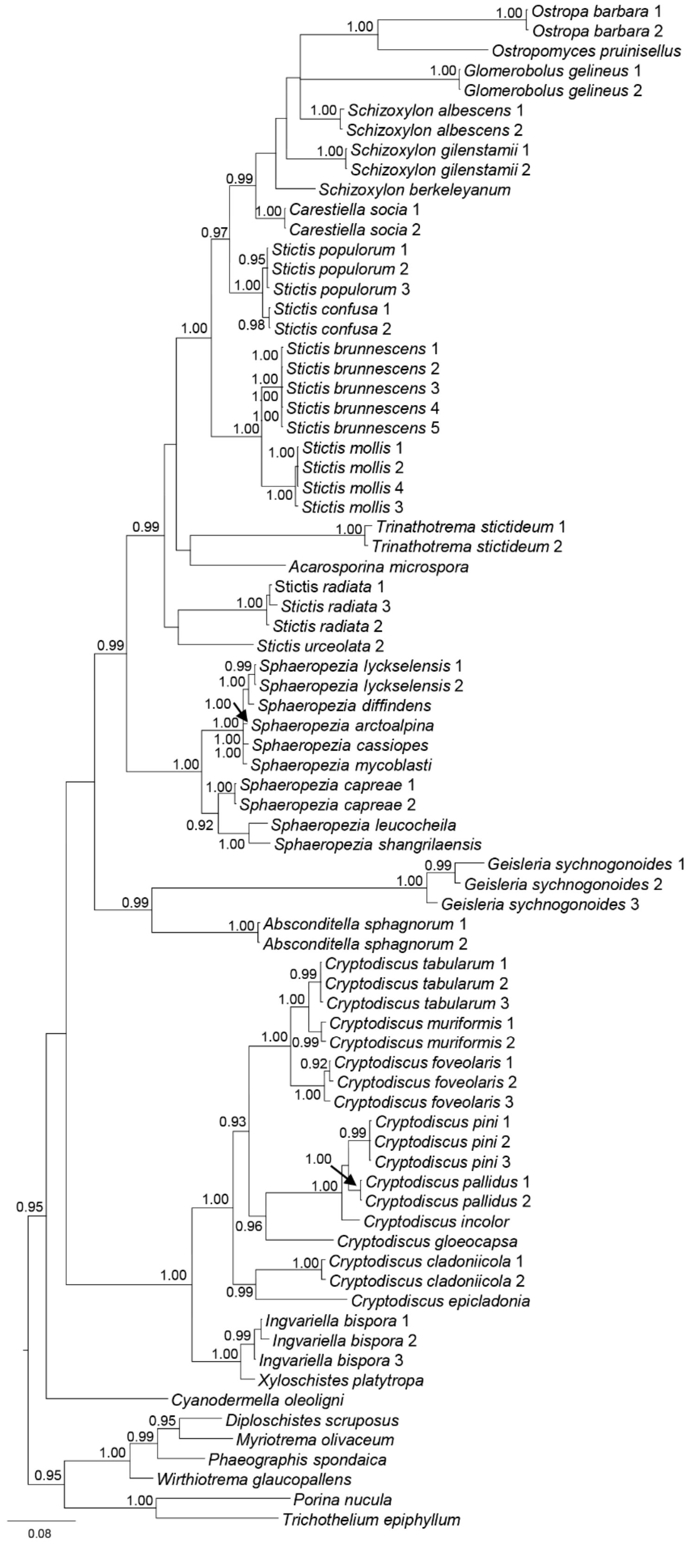

Figure S5 Best-scoring RAxML tree reconstructed based on analysis of a single dataset of LSU sequence data. Bootstrap support values for BP equal to or greater than 0.90 is defined above the nodes.

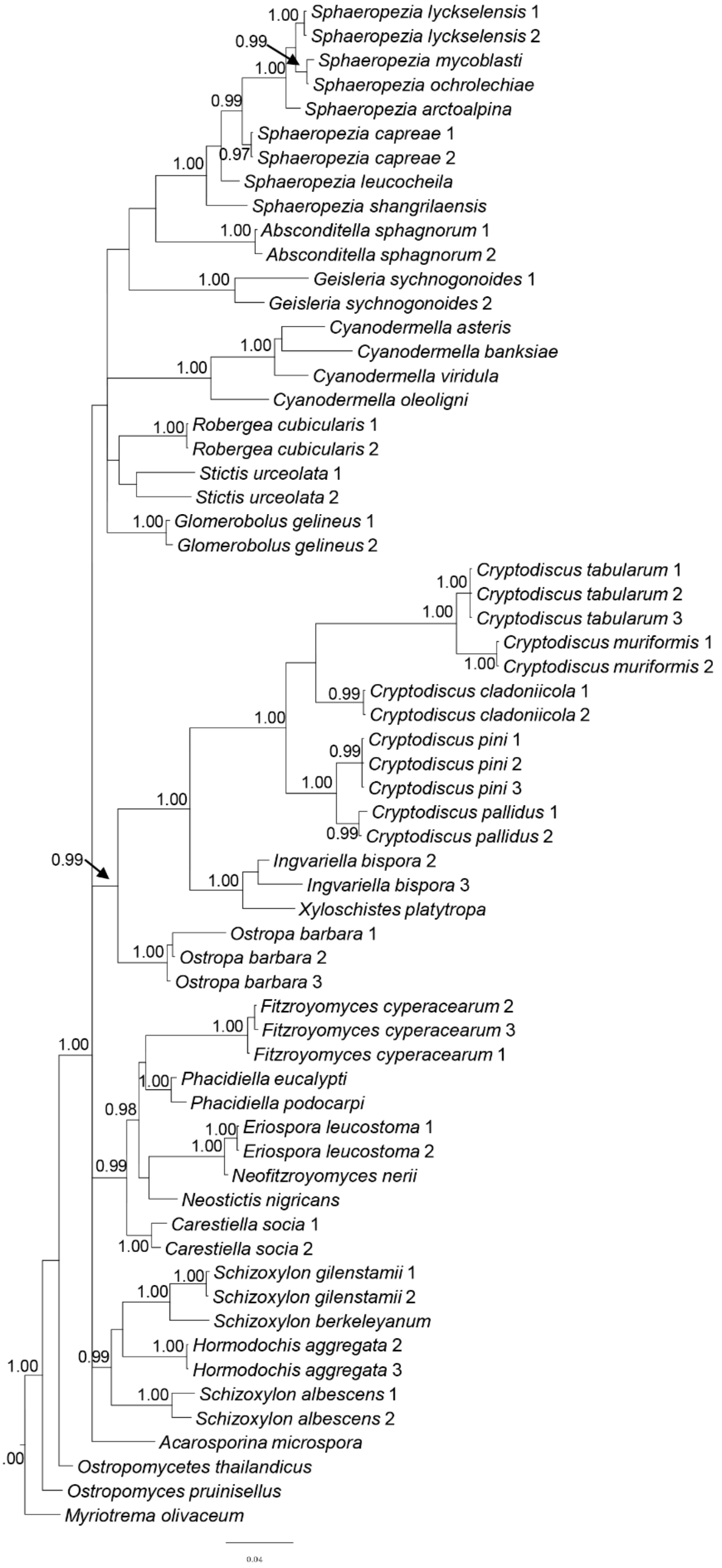

Figure S6 Best-scoring RAxML tree reconstructed based on analysis of a single dataset of ITS sequence data. Bootstrap support values for BP equal to or greater than 0.90 is defined above the nodes.

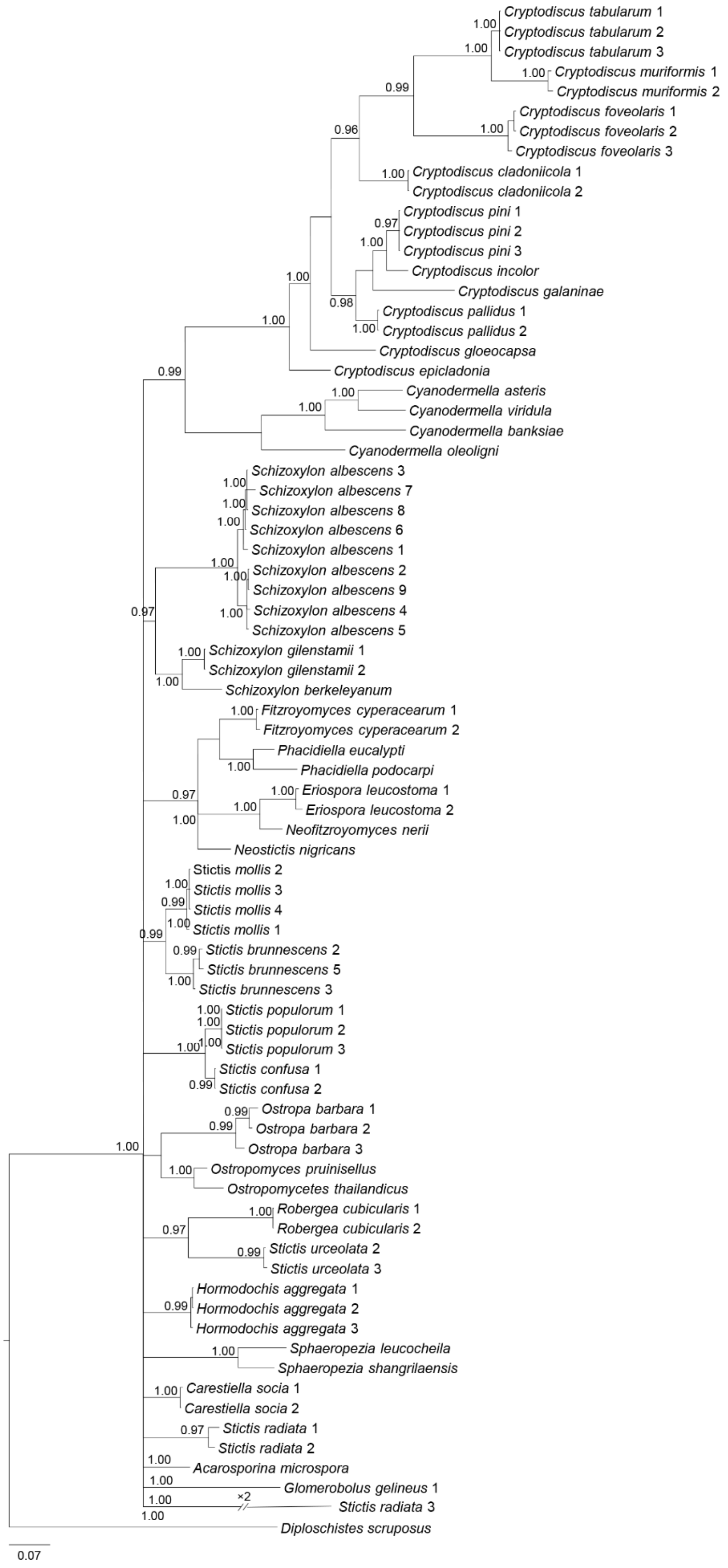

Supplement: Supplementary file 1 [file jof-07-00105-s001.pdf]
